# Intra-patient potassium variability after hypothermic cardiac arrest: a multicentre, prospective study

**DOI:** 10.1186/s13049-019-0694-3

**Published:** 2019-12-16

**Authors:** M. Pasquier, M. Blancher, S. Buse, B. Boussat, G. Debaty, M. Kirsch, M. de Riedmatten, P. Schoettker, T. Annecke, P. Bouzat

**Affiliations:** 1 Emergency Department, Lausanne University Hospital, Lausanne, Switzerland; 2 Department of Emergency Medicine, SAMU 38, University Hospital of Grenoble Alps, Grenoble, France; 3 Department of Cardiac Surgery, Lausanne University Hospital, Lausanne, Switzerland; 4 Emergency Department, Sion Hospital, Sion, Switzerland; 5 Department of Anesthesiology, Lausanne University Hospital, Lausanne, Switzerland; 6 Klinik für Anästhesiologie und Operative Intensivmedizin, University Hospital of Cologne, Köln, Germany; 7 Department of anesthesiology and critical care, Grenoble Alps Trauma Center, University Hospital of Grenoble, Grenoble, France

**Keywords:** Cardiac arrest, ECMO, ECPR, Gasometer analyser, hypothermia, accidental, Potassium, Resuscitation, Triage

## Abstract

**Background:**

To date, the decision to set up therapeutic extra-corporeal life support (ECLS) in hypothermia-related cardiac arrest is based on the potassium value only. However, no information is available about how the analysis should be performed. Our goal was to compare intra-individual variation in serum potassium values depending on the sampling site and analytical technique in hypothermia-related cardiac arrests.

**Methods:**

Adult patients with suspected hypothermia-related refractory cardiac arrest, admitted to three hospitals with ECLS facilities were included. Blood samples were obtained from the femoral vein, a peripheral vein and the femoral artery. Serum potassium was analysed using blood gas (BGA) and clinical laboratory analysis (CL).

**Results:**

Of the 15 consecutive patients included, 12 met the principal criteria, and 5 (33%) survived. The difference in average potassium values between sites or analytical method used was ≤1 mmol/L. The agreement between potassium values according to the three different sampling sites was poor. The ranges of the differences in potassium using BGA measurement were − 1.6 to + 1.7 mmol/L; − 1.18 to + 2.7 mmol/L and − 0.87 to + 2 mmol/L when comparing respectively central venous and peripheral venous, central venous and arterial, and peripheral venous and arterial potassium.

**Conclusions:**

We found important and clinically relevant variability in potassium values between sampling sites. Clinical decisions should not rely on one biological indicator. However, according to our results, the site of lowest potassium, and therefore the preferred site for a single potassium sampling is central venous blood. The use of multivariable prediction tools may help to mitigate the risks inherent in the limits of potassium measurement.

**Trial registration:**

ClinicalTrials.gov Identifier: NCT03096561.

## Background

Accidental hypothermia, which is often caused by homelessness or substance abuse, mountaineering accidents or suicide attempts, is a condition associated with significant mortality [1, 2]. However, patients with accidental hypothermic cardiac arrest (CA) may have an excellent neurological outcome after extracorporeal life support (ECLS) rewarming [3, 4]. The critical decision to initiate rewarming of a CA patient must be made rapidly, based on a very limited amount of information and ancillary examination, while the patient is undergoing cardiopulmonary resuscitation (CPR). For clinicians, it is challenging to distinguish between hypothermic cardiac arrest that would benefit from ECLS and a cold, dead person for whom resuscitation would be futile. According to international recommendations, the decision to initiate ECLS for hypothermic CA patients is essentially based on the serum potassium level, since a high serum potassium value is associated with a poor outcome in these patients [1, 2, 5–10].

Despite the well-established role of serum potassium in the triage of hypothermic CA victims [1, 8–10], no recommendation exists regarding the site (arterial, venous, central or peripheral) of blood sampling for potassium measurement. Similarly, the type of analytical technique (e.g. by blood gas analysis [BGA] or by the hospital central laboratory) is also not specified. Variation in potassium concentration may, however, occur depending on the site of sampling due to local factors [11–14], or measurement technique [15–18]. Improving our knowledge on this topic is of the utmost importance. Indeed, if a life-or-death decision has to be made based on a single biological indicator, this measurement should be perfectly reliable. Our goal was to study the potassium concentration at different sampling sites (central venous, peripheral venous and arterial) and by different analytical methods (gasometry and laboratory) among hypothermic CA patients. Our hypothesis was that potassium concentrations will vary depending on the sampling site but also on the analytical method used.

## Methods

### Patients

We included consecutive adult patients with refractory CA related to a suspicion of deep hypothermia (core temperature < 30 °C at hospital admission) from November 2016 to February 2019. We excluded pregnant women and patients with traumatic cardiac arrest. The participating centres were two tertiary care university hospitals (Grenoble, France and Lausanne, Switzerland) and one non-university trauma centre (Sion, Switzerland). The three hospitals have ECLS rewarming capacities. The study was approved by the institutional review board of the participating centres (CER-VD N° 2016 01760 and ID-RCB: 2016-A01762–49). This study was registered in the ClinicalTrials.gov registry (Identifier: NCT03096561).

### Hospital management of patients

Patients were included at hospital admission. Core temperature was measured in the lower third of the oesophageus [1, 8] to confirm hypothermia < 30 °C. Blood samples were obtained at hospital admission from the following three sites: the femoral vein, the femoral artery and a peripheral vein. Central (femoral) samples were obtained either under ultrasound guidance while interrupting CPR or through intravascular catheters. Blood samples were analysed by the following two different analytic methods: BGA and by the hospital central laboratory (CL). The lowest value of the three potassium samples analysed by BGA was used in the decision process to qualify or disqualify patients for ECLS rewarming.

### Biological sampling and analysis

We used heparinized syringes (BD A-line™) to collect whole blood from each of the three sampling sites for BGA (ABL-800, Radiometer). Potassium was measured using a selective-ion electrode (potentiometry). We also collected the following results: lactate, pH, PaCO_2_ and PaO_2_. BGA is available in the emergency department and allows immediate determination of potassium concentration at the bedside. For CL analysis, blood samples were collected in 2 different sample tubes (BD Vacuitainer® - SST™ II and BD - LH PST II). Potassium was measured using indirect potentiometry (VISTA® SIEMENS V-LYTE® integrated multisensor technology - PN797800.002 Rev. D - Siemes healthcare diagnostic - 2013). We also measured pH and lactate. During the study period, the BGA and CL analysers were checked and calibrated regularly according to the manufacturer’s recommendations. The time of each of the biological samplings was recorded.

### Data collection

The following prehospital variables of interest were collected for each patient when available: age, gender, prehospital core temperature, whether or not CA was witnessed, time of CA, if asphyxia was associated, first documented cardiac rhythm after CA (e.g. asystole, ventricular fibrillation, pulseless ventricular tachycardia and pulseless electrical activity (PEA)) and prehospital treatments administered (e.g. defibrillation, reanimation drugs). The time of hospital admission and first oesophageal temperature (°C) were collected. When relevant, the modality of hospital rewarming, the no-flow and low-flow duration, as well as the time of ICU and hospital discharge were collected. Patient outcomes were assessed by survival and neurological status at hospital discharge and at 3 months. The neurological outcome was assessed by the Cerebral Performance Category (CPC) [19]. A CPC of 1 or 2 was considered as a “favorable neurological outcome” [4, 20, 21].

### Outcomes

Our primary outcome was the comparison between central venous and peripheral venous potassium by BGA. Secondary outcomes were the comparison between venous and arterial potassium and the comparison between BGA and CL analysis.

### Statistical analysis

The sample size was calculated to detect a difference of at least 1 mmol/L in the average potassium concentration between central and peripheral venous samples. Using an estimated standard deviation of 3.5, a correlation rate of 95%, an alpha risk level of 0.05 and a power of 80%, the total number of patients was estimated at 12. Cases with missing data regarding the primary outcome (central and/or peripheral venous potassium) in both laboratory and gasometry samples were excluded from the comparative analysis. When BGA data were missing (peripheral or central venous potassium rate), they were replaced by CL data. Descriptive statistics were determined for variables of interest collected from all included patients and expressed as frequencies, means and standard deviations, or medians and interquartile ranges (IQR). Comparisons were conducted by using the Student’s *t*-test or the Wilcoxon rank-sum test for appariated data as appropriate, to compare potassium levels between 1) central and peripheral venous sites, 2) venous and arterial sampling and 3) laboratory and gasometry analysis. The concordance between the groups including these individual data was evaluated graphically using Bland and Altmann graphs [22]. A bilateral *P* value of < 0.05 was considered to indicate a significant difference between patient groups. Data were retrieved from the patient information database that was established for this study and exported into Stata Special Edition version 14.0 (Stata Corporation, College Station, TX, USA) for analysis.

## Results

**Fig. 1:**
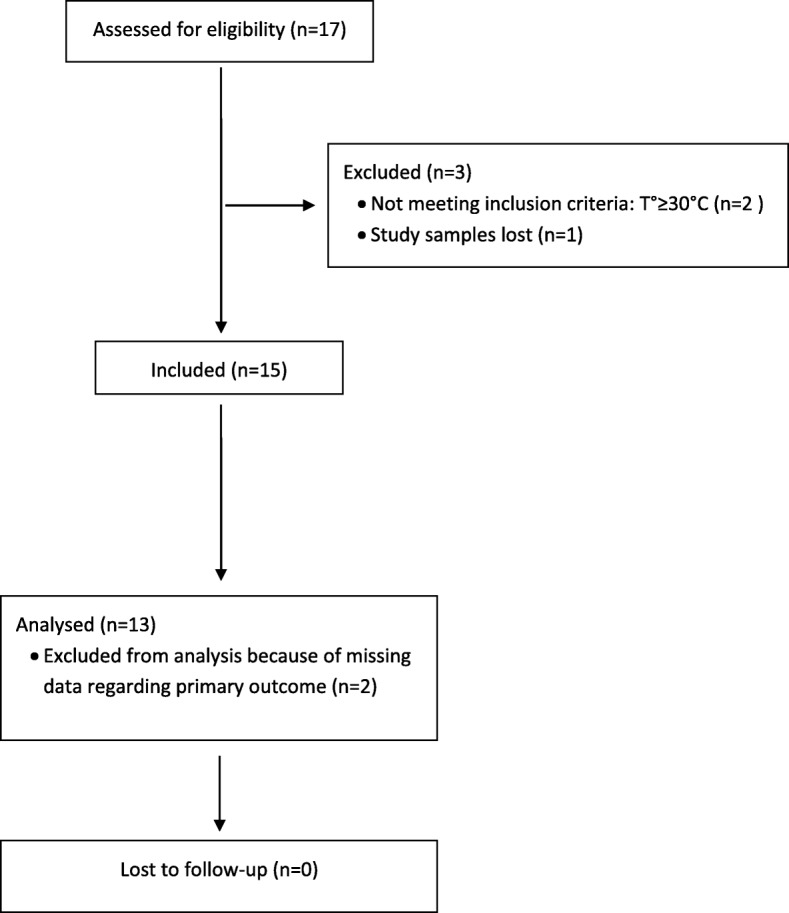
Flow chart of study patients

Within the study period, we enrolled 15 consecutive patients. Two cases were exluded from the analysis because of missing data regarding the primary outcome (Flow chart in Fig. 1). Overall characteristics of the included patients are presented in Table 1. The mean age was 53 ± 17 years (range 25–87), and 60% of the patients were male (*n* = 9). CA was witnessed in 33% (*n* = 5). The mean prehospital oesophageal temperature was 22.1 ± 5.8 °C (range 8–28.3), and the mean hospital oesophageal temperature was 22.0 ± 5.1 °C (range 10.7–28.3). Median arterial lactate was 8.60 mmol/L (IQR 4.9–12.92, range 2.2–22.5). Eleven of the 15 patients (73%) underwent ECLS: 8 patients had hospital return of a spontaneous circulation (ROSC), and 5 patients survived to hospital discharge (33%) with a good neurological outcome (3-month CPC of 1) for all 5 survivors.

**Table 1:** Overall characteristics of the included patients (*n* = 15). *A* asystole, *CA* cardiac arrest, *CPC* Cerebral Performance Category, *CPR* cardiopulmonary resuscitation, *ECLS* extracorporeal life support, *F* female, *M* male, *NA* not available (including haemolysis), *PEA* pulseless electrical activity, *ROSC* return of spontaneous cardiac circulation, *VF* ventricular fibrillation

Patient	#1	#2a	#3	#4a	#5b	#6	#7	#8	#9	#10	#11b	#12	#13	#14	#15
Age	87	32	44	52	57	45	53	41	76	74	49	64	25	35	61
Gender	F	F	M	M	F	M	M	F	M	M	M	M	M	F	F
Asphyxia CA^a^	No	No	No	Yes	No	No	No	NA	No	No	No	No	Yes	No	No
No-flow duration (min)	0	10	0	180	0	NA	NA	NA	NA	1	5	0	18	0	0
Initial core temperature (°c)	25.0	24.0	25.0	24.7	24.1	11.0	8.0	25.6	17.0	26.0	25.0	19.9	24.0	24.3	28.3
First recorded rhythm	VF	A	VF	NA	VF	A	A	NA	A	A	VF	A	A	PEA	VF
CPR duration (low flow min)	10	78	215	38	43	NA	NA	165	99	54	145	117	87	45	55
Blood gas analyser															
Central venous potassium	3.5	NA	4.5	NA	NA	10.0	14.6	5.9	9.2	2.2	4.9	3.5	4.2	2.6	2.7
Peripheral venous potassium	4.25	NA	4.9	NA	3.0	10.4	13.0	6.4	8.4	3.9	NA	3.6	5.1	2.6	3.7
Arterial potassium	3.70	14.80	4.5	11.2	4.6	11.8	15.0	5.5	9.8	4.9	3.7	4.2	NA	2.5	2.9
pH	7.3	6.4	6.9	NA	7.1	7.0	6.9	NA	6.9	7.0	7.1	7.2	NA	7.2	7.3
Arterial lactates	11.2	22.5	3.5	16.1	4.9	12.9	8.6	18.2	9.6	5.0	8.6	6.9	NA	3.7	2.2
Central laboratory															
Central venous potassium	3.4	NA	NA	NA	3.8	11.3	NA	6.4	9.0	2.1	4.7	3.3	4.2	3.1	3.1
Peripheral venous potassium	4.6	NA	NA	NA	4.2	NA	17.2	6.4	9.1	2.3	3.4	3.5	5.5	3.0	3.7
Arterial potassium	4.0	NA	NA	NA	4.9	13.3	19.0	6.4	10.3	4.3	4.8	4.2	NA	3.0	3.3
Outcome															
ECLS	No	No	Yes	No	Yes	Yes	No	Yes	Yes	Yes	Yes	Yes	Yes	Yes	Yes
ROSC	Yes	No	Yes	Yes	No	Yes	No	No	Yes	Yes	No	Yes	Yes	Yes	No
Survival at hospital discharge	No	No	No	No	No	No	No	No	No	No	Yes	Yes	Yes	Yes	Yes
CPC at 3 months for survivors	NA	NA	NA	NA	NA	NA	NA	NA	NA	NA	1	1	1	1	1
HOPE Score	96%	17%	28%	1%	96%	NA	NA	NA	13%	NA	54%	74%	22%	98%	85%

^a^cases not eligible for analysis of primary outcome because of missing data

^b^cases for which missing data for blood gas analysis of either central or peripheral potassium were replaced by central laboratory data

A summary of the potassium values by sampling site and analytic method used is provided in Table 2**.** The ranges of the differences in potassium for the 3 different sites using BGA measurement were − 1.6 to + 1.7 mmol/L; − 1.18 to + 2.7 mmol/L and − 0.87 to + 2 mmol/L when comparing, respectively, central venous and peripheral venous (*n* = 13 patients), central venous and arterial (*n* = 12 patients), and peripheral venous and arterial potassium (n = 12 patients).

**Table 2:** Summary of potassium values according to the sampling sites and analytical method used

	Median (IQR)	Mean ± SD	Range
Blood gas analyser			
Central venous (*n* = 12)	4.36 (3.09–7.57)	5.66 ± 3.76	2.2–14.6
Femoral artery (*n* = 14)	4.75 (3.71–11.2)	7.09 ± 4.46	2.5–15
Peripheral venous (*n* = 12)	4.55 (3.65–7.4)	5.77 ± 3.23	2.6–13
Central laboratory			
Central venous (*n* = 11)	3.8 (3.1–6.4)	4.95 ± 2.84	2.1–11.3
Femoral artery (*n* = 11)	4.8 (4.04–10.3)	7.05 ± 5.08	3–19
Peripheral venous (*n* = 11)	4.2 (3.4–6.4)	5.72 ± 4.25	2.3–17.2

**Fig. 2:**
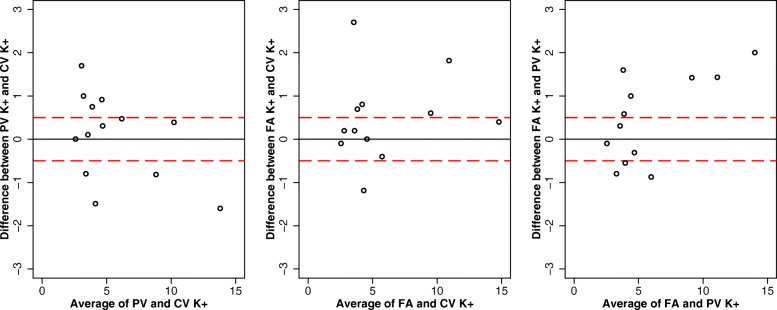
Bland-Altman analysis of the difference in potassium between the three sampling sites when analysed by blood gas analyser. For each comparison, the mean value between the two sites is plotted against their difference. Red dash lines represent the ±0.5 mmol/L upper and lower limits of standard criteria for acceptable performance for potassium. CV = central vein; FA = femoral artery; K + =potassium; PV = peripheral vein

When considering BGA analysis, on average, there was a trend for central venous potassium to be lower than peripheral potassium (median difference of 0.31, IQR -0.8 − + 0.75), but this difference was not statistically significant (Wilcoxon signed-rank test *p* = 0.6245). On average, the arterial potassium was higher than both the central and peripheral venous potassium (median difference of, respectively, 0.3 mmo/L (IQR -0.05 − + 0.745) and 0.445 mmol/L (IQR -0.43 − + 1.425), but these differences were not statistically significant (*P* value for Wilcoxon signed-rank test of, respectively, 0.0914 and 0.1361). The Bland-Altman analysis of the difference between the 3 sampling sites is presented in Fig. 2. There was no agreement between potassium values according to the 3 different sampling sites using BGA.

**Fig. 3:**
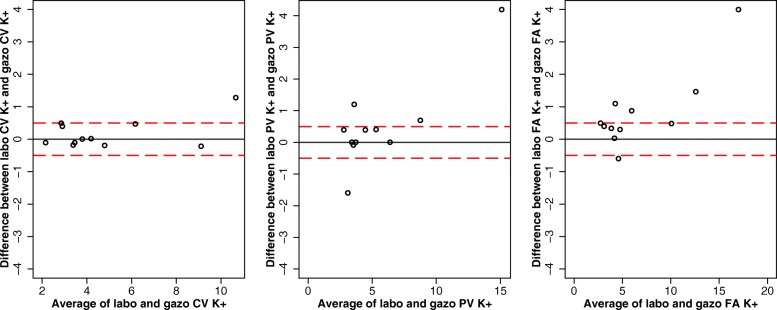
Bland-Altman analysis of the difference in potassium between the two analytic methods for each of the three sampling sites. For each comparison the mean value of potassium for each of the two analytic methods (blood gas analyser or central labolatory) is plotted against their difference. Red dash lines represent the ±0.5 mmol/L upper and lower limits of standard criteria for acceptable performance for potassium. The mean difference in potassium obtained from the central vein between the blood gas analyser and central labolatory was 0.81 (*p* = 0.0208). The mean difference in potassium obtained from the peripheral vein between the blood gas analyser and central labolatory was 0.51 (*p* = 0.1491). The mean difference in potassium obtained from the artery between the blood gas analyser and central labolatory was 0.17 (*p* = 0.5042). BGA = blood gas analyser; CL = central laboratory (CL); K + =potassium

When considering CL analysis, arterial potassium was significantly higher than central or peripheral venous potassium, as no significant difference was found between central and peripheral venous potassium (Additional file 1). When comparing the two analytic methods (BGA and CL), we found no significant difference regarding central venous, peripheral or arterial potassium values (Fig. 3).

## Discussion

The average difference in potassium concentration between central and peripheral venous samples was lower than 1 mmol/L in our prospective study of hypothermic CA patients. However, there was no agreement between potassium values according to the three different sites of sampling using BGA. Our study may have some important clinical implications.

### Clinical implications of the differences found in potassium concentration between sampling sites

Potassium is one of the main triage tools, and the only biological marker used in clinical practice when deciding to rewarm a hypothermic CA patient [1, 8–10]. Hypothermia initially causes a decrease in the potassium level, due to an intracellular shift [23]. The presence of hyperkalaemia in deep hypothermia is a sign of irreversible cellular lysis and of a poor prognosis [5, 6, 23]. The highest potassium rate in a survivor of accidental hypothermia was 11.8 mmol/L, in a child [24]. The highest recorded levels of potassium were 9 mmol/L [25] and 6.4 mmol/L in an adult who was successfully resuscitated from hypothermic CA and an avalanche victim, respectively [26]. A potassium level < 12 mmol/L in a hypothermic CA patient usually indicates ECLS rewarming [1]. In contrast, with a potassium level ≥ 12 mmol/L, cessation of reanimation efforts should be considered [1, 9, 10]. Lower potassium cut-offs have been proposed for avalanche victims, namely 8 mmol/L or, more recently, 7 mmol/L [1, 8, 10, 27, 28]. According to our results, the observed differences in potassium analysed by BGA between the different sites of sampling would not have reclassified a single patient if the decisions to qualify a CA patient for ECLS rewarming were based on the potassium cut-offs of 7, 8 or 12 mmol/L. Only one patient would have been classified differently according to the sampling site when considering CL analysis. This patient had an arterial potassium of 13.3 mmol/L and central venous potassium of 11.3 mmol/L, which would have resulted in denying ECLS to this patient if a cut-off of 12 mmol/L arterial potassium had been applied.

The proposed standard criteria for acceptable performance for potassium is the target of ±0.5 mmol/L [16, 29]. A difference of 1 mmol/L may be clinically relevant and influence patient management options [15]. Such commonly accepted values may be revised to lower figures in the specific setting of hypothermic CA. In the latter situation, potassium has been used for decades in a dichotomous way to distinguish between ECLS indication (and potential for survival with good neurological outcome) and death. The maximal difference between, respectively, central venous and peripheral venous potassium measurements was 1.7 mmol/L, which is far above this threshold. Moreover, our limits of agreement between central and peripheral venous potassium were − 1.8 and + 2.0 mmol/L, which is far broader than the standard criteria of ±0.5 mmol/L. The clinical consequence would be to underestimate the patient’s potential for survival. Practically speaking, if using the actual dichotomous potassium triage, this would mean denying a patient with the potential to survive access to ECLS rewarming. This is especially important in the situation of hypothermic CA patients, for whom this would signify the death of the patient, as survivors following ECLS rewarming have much better neurological outcomes than survivors of normothermic CA [30]. Based on the important variations in the potassium level we found in this study within the same patient, we assume that the potassium level is not reliable as a single indicator for decision-making.

Life-or-death decisions based on a single parameter, much less a biological one, are rare in medicine, and our results underline the fragility and confirm the inadequacy of the “potassium-only” triage of hypothermic CA patients [31]. Our study results may therefore directly influence practice in several ways. First, if only one sampling site is used, then central venous sampling should be preferred. Also not statistically significant, the − 0.31 mmol/L difference in central venous potassium compared with peripheral potassium may represent a difference that is no longer considered acceptable [32], and that may be clinically significant in the very specific setting of hypothermic CA. Another reason to choose central venous potassium may be that, although peripheral sampling may avoid some complications linked to central blood sampling, such as iatrogenic trauma to the femoral vessels [33], it may be extremely technically challenging to obtain a peripheral sample, as shown by some of our study cases. Given the relative ease of obtaining femoral compared with peripheral venous blood sampling as well as the trend for central venous potassium to be lower than the peripheral one, we would propose to use central venous blood as the preferred site for a single potassium sampling. Another solution may be to perform multisite potassium sampling and decide using the lowest value of potassium. The important differences we found in both directions between sampling sites may support this latter proposal. Whatever potassium value is used, all has to be done to limit pseudohyperkalaemia, which would cause a falsely elevated potassium, possibly leading to inappropriate therapeutic decisions. Finally, the use of a multivariable dedicated tool like the HOPE score to guide decision-making should be promoted to increase the reliability of the decision to administer ECLS rewarming [4].

### Origin of the differences in potassium values between the different samplings

The variations found in potassium concentration depending on the site of sampling may be explained by several reasons. First, differences may occur due to local factors [11–14]. This may arise in cases of hypothermic CA, where phenomena like peripheral ischemia, crush syndrome due to immobilization, or circulation shifts may influence the local potassium concentration, notably between peripheral (accumulation) and central blood. Another reason may be pseudo-hyperkalaemia, which may also occur during or after blood collection, due to excessive tourniquet time or mechanical trauma during venepuncture or transport [13, 14, 34]. This may be the case especially in hypothermia, where the puncture or catheterization of vessels is often difficult, notably due to the difficulty in identifying a pulse in cardiac arrest patients [33] and where numerous attempts to puncture the vessels are common [33]. The use of ultrasound, which has been recommended to prevent this [33], or sampling through an in-place femoral catheter, was designed not only to ensure the correct sampling site but also to minimize the risk of haemolysis. In contrast, the fact that peripheral venous samples were not obtained in some cases because of technical difficulties may signify problems with these samples, which may have more elevated potassium through haemolysis, which has been shown to be especially prevalent in the emergency department [35].

### Clinical implications of the differences found in potassium concentration between analytical methods

The type of analytical technique for potassium measurement (BGA or CL) is not specified in the recommendations. Some studies showed that potassium was lower if measured by BGA than if measured by CL [15, 17, 18]. Like other authors, we were unable to find any significant difference in potassium measurement by BGA compared with CL in our study [36]. This point might, however, not be clinically very relevant in such emergency situations, where blood samples and BGA results are obtained in a few minutes, which is especially attractive or important in clinical scenarios where time is of the utmost importance [17, 18]. Although in hypothermia low flow duration is less important than in normothermic CA, short low flow is an independent factor for survival in hypothermic CA, and waiting for CL analysis is not even an option to consider [4]. Our results underline the reliability of BGA compared with laboratory analysis. As we found no significant difference between the two methods, BGA results can be considered valid for a value which should under no circumstances be subjected to variation.

### Limitations

Our study suffers from several limitations. First, potassium measurement may be subject to important preanalytical bias [36]. Some of these biases – notably haemolysis of blood samples taken during CPR – may be especially important in the specific setting of hypothermic CA, where blood sampling is often difficult, notably due to the difficulty in identifying a pulse in these patients [33]. We did not plan to control – as suggested by some [36] – haemolysis in our study, notably because under real-life conditions this would not be feasible given the delay in obtaining results from the CL. However, we tried to minimize this bias regarding central sampling by the following methods that may be used in similar situations: sampling through an in-place catheter, or by vessel puncture under visual or ultrasonographic control, including by momentarily stopping reanimation. Second, we did not specify the drawing method for peripheral blood sampling. It has been showed that the risk of haemolysis is higher when the sample originates from an intravenous catheter compared with blood drawn by a straight needle [35]. However, direct puncture is often considered impractical in settings like the ED, where intravenous catheters are placed quasi-systematically and their use is recognized as virtually unavoidable [35, 37]. Furthermore, in our setting most patients arrive at the hospital with a peripheral catheter already placed by the emergency medical services (EMS).

## Conclusions

In a cohort of consecutive hypothermic CA patients, we did not find an average difference in potassium concentration higher than 1 mmol/L between central and peripheral venous samples. However, we found important and clinically significant variability in potassium values between central venous, peripheral venous and arterial sampling sites. Our results suggest that the site of lowest potassium, and therefore the preferred site for a single potassium sampling is central venous blood. Analysis of serial samplings from different sites may be an alternative, if clinically feasible. Preanalytical considerations are important, to lower the risk of bias (falsely elevated potassium), which might signify, in the specific case of a hypothermic CA patient, the unjustified cessation of reanimation efforts. BGA is a fast and reliable tool that should be preferred to the CL in hypothermic CA. The use of multivariable prediction tools like the HOPE score may help mitigate the risks inherent in the limits of potassium measurement.

## Supplementary information

**Additional file 1.** Bland-Altman analysis of the difference in potassium between the three sampling sites when analysed by the central laboratory. For each comparison the mean value of potassium for each of the two sites is plotted against their difference. Red dash lines represent the ±0.5 mmol/L upper and lower limits of standard criteria for acceptable performance for potassium. The mean difference was 0.26 (*p* = 0.153), when comparing central venous and peripheral potassium. The mean difference was 0.83 (*p* = 0.0143), when comparing central venous and arterial potassium. The mean difference was 0.68 (*p* = 0.0511when comparing peripheral venous and arterial potassium. BGA = blood gas analyser; CL = central laboratory (CL); CV = central vein; FA = femoral artery; K + =potassium; PV = peripheral vein.

## Data Availability

The datasets generated and/or analysed during the current study are not publicly available but are available from the corresponding author on reasonable request.

## Abbreviations

BGA: Blood gas analysis
CA: Cardiac arrest
CL: Clinical laboratory analysis
CPC: Cerebral Performance Category
CPR: Cardiopulmonary resuscitation
ECLS: Extra-corporeal life support
EMS: Emergency medical services
IQR: Interquartile ranges
PEA: Pulseless electrical activity
ROSC: Return of a spontaneous circulation
